# The study of neuroprotective effects and underlying mechanism of Naoshuantong capsule on ischemia stroke mice

**DOI:** 10.1186/s13020-020-00399-7

**Published:** 2020-11-17

**Authors:** Lvkeng Luo, Shuling Wu, Ruiqi Chen, Hongyu Rao, Wei Peng, Weiwei Su

**Affiliations:** Guangzhou Quality R&D Center of Traditional Chinese Medicine, Guangdong Key Laboratory of Plant Resources, School of Life Sciences, Sun Yat-Sen University, Guangzhou, 510275 People’s Republic of China

**Keywords:** Naoshuantong capsule, Network pharmacology, Stroke, Neuroprotective, Mice

## Abstract

**Background:**

Naoshuantong capsule (NSTC) is an oral Chinese medicine formula composed of Typhae Pollen, Radix Paeoniae Rubra, Curcumae Radix, Gastrodiae Rhizoma and Radix Rhapontici. It has been widely used at the acute and recovery stage of ischemic stroke since 2001. Comparing with its wide clinical application, there are only few studies emphasize on investigating its pharmacological effects.

**Methods:**

To more generally elucidate the underlying mechanisms in this study, we identified active ingredients in NSTC by a network pharmacology approach based on transcriptomics analysis and pharmacological experiments. Modified neurological severity scores and morphometric analysis using Nissl staining were employed to evaluate the neuroprotective effects of NSTC on ischemia stroke in mice.

**Results:**

The results showed that NSTC had preventive and protective effects on ischemia stroke, featuring repair of brain tissue during the sub-acute stage of stroke. This may attribute to the underlying mechanisms including anti-inflammatory, antioxidant, and anti-apoptotic activities, as well as an attenuation of excitatory amino acids (EAAs) toxicity of the active ingredients, especially the most active apigenin, from NSTC. Specifically, naringenin, calycosin, gastrodin, caffeic acid, paeoniflorin, and *β*-elemene seem to be also pharmacological active substances responsible for the anti-inflammatory effects. Meanwhile, 13-hydroxygemone, gastrodin, and *p*-hydroxybenzyl alcohol contributed to the attenuation of EAAs toxicity Furthermore, apigenin, naringenin, calycosin, gastrodin, and *β*-elemene accelerated the repair of brain ischemic tissue by up-regulating the expression of TGF-β1 levels.

**Conclusions:**

The present study identifies the active ingredients of NSTC and illustrates the underlying mechanism using a combination of network pharmacology, transcriptomics analysis, and pharmacological experiments.

## Background

Stroke, as an important cadio-cerebrovascular disease, is one of the leading causes of death and disability in adults across the world. According to estimates of the national morbidity in 2018, more than 13 million people in China suffer a stroke [1]. Stroke can be divided into two categories: ischemic and hemorrhagic stroke. Ischemic stroke is more prevalent in China, accounting for 69.6% of the new stroke patients, than hemorrhagic stroke in China in 2017 [2]. Its prevention and treatment strategies must be modified and upgraded as ischemic stroke have emerged as a social problem that needs to be solved urgently.

Traditional Chinese medicine (TCM) has a relatively complete theoretical system in the treatment of stroke with its unique advantages after thousands of years of accumulation in clinical experience. The 6-component oral TCM formula *Naoshuantong* capsule (NSTC) is an oral Chinese medicine formula composed of Typhae Pollen (TP), Radix Paeoniae Rubra (PR), Curcumae Radix (CR), Gastrodiae Rhizoma (GR) and Radix Rhapontici (RR) was approved by the China Food and Drug Administration (CFDA) as a clinical drug for stroke in 2001. NSTC is mainly used during the acute period and body recovery after cerebral ischemia in the clinic at present. NSTC has been reported to effectively restore the neurological function [3] with favorable recuperation in patients after stroke [4]. Molecular evidences show that NSTC exerts an inhibitory effect on neuronal apoptosis caused by stroke [5], with improved blood rheology and energy metabolism in rats [6]. Although the constituents [7] and metabolic profile [8] of NSTC have been fully investigated, not much research has been done in pharmacological mechanism of NSTC, which is an emerging bottleneck restraining its clinical application.

The concept of network pharmacology since its first proposal in 2007, mainly aims at constructing a complex biological network of “disease-gene-target-drug” interactions by integrating data. The biggest superiority of network pharmacology is that it breaks through the limitations of previous research models which focus on a single drug, a single target, and a single disease. It is a more holistic, systematic scientific thought and research strategy, which underpins for more profound on TCM [9]. Meanwhile, transcriptomics also plays a unique role in investigating mechanisms behind the effects of the TCM formulas and subtle compatibility of TCM [10].

In the present work, to evaluate the preventive and protective effects of NSTC on stroke, modified neurological severity scores (mNSS) test and Nissl staining were applied to detect neurological deficits and ischemic damages in photothrombotic stroke mice respectively. Then we performed a network pharmacology approach combining with transcriptomics analysis and pharmacological experiments to identify the active components and explore the putative pharmacological mechanisms behind NSTC’s roles in alleviating the brain injury in C57BL/6 mice.

## Materials and methods

### The preparation of NSTC

NSTC (batch No.: 160510) were provided by Guangdong Huanan Pharmaceutical Co., Ltd. (Dongguan, China). The product (TP, 33.33%; PR, 23.78%; CR, 19.10%; GR, 9.55%; RR, 14.23%) was produced according to the 2015 Chinese Pharmacopoeia (CP) as follows. 1) Ethanol (70%) was added to PR which was heated and extracted twice for 1 h each time. Then the extracts were combined and filtered after ethanol was recovered from the filtrates, and the mixture was concentrated to an appropriate amount, dried, pulverized, and added with an appropriate amount of calcium hydrogen phosphate. They were mixed and dried for later use. CR was added with 80% ethanol and heated twice under reflux for 1 h each time. The mixed extracts were filtered for further use. The dregs of PR and CR, TP (confined in gauze bags), GR, and RR were boiled in water twice, 1 h each time, to obtain decoction which was then filtered, and the filtrate was concentrated to a clear paste with a relative density of 1.04 to 1.10 (40 °C). The ethanol was added to make the alcohol content reach 70%; Afterward, the supernatant was collected and mixed with the alcohol extract of CR. After ethanol was recovered, the mixture was concentrated to an appropriate amount, dried, crushed, and added with an appropriate amount of calcium hydrogen phosphate. After PR dry powders were added, it was granulated with hypromellose ethanol solution, dried, and mixed with talc, silicon dioxide and magnesium stearate to make capsules.

### Standardization of NSTC

The constituent analysis of NSTC was carried out by mass spectrometry (MS). NSTC (0.5 g) was put in a container for a 30-min methanol (10 mL) bath at 40 kHz twice. The extract solution (1 mL) was filtered through a 0.22 μm membrane before analysis. The reference standards of typhaneoside, gallic acid, gastrodin, catechin, chlorogenic acid, β-elemene, paeoniflorin, protocatechuate, apigenin, β-ecdysterone, isorhamnetin-3-O-neohespeidoside (National Institute for the Control of Pharmaceutical, China); calycosin, naringenin (Sigma, St. Louis, USA) were dissolved in methanol at the concentration of 0.1 mg/mL for each compound to make the mixed standard solution.

The UPLC-Trip-TOF–MS/MS analysis was performed by the Triple TOF-TM 5600 plus (AB SCIEX, Foster City, CA) hybrid triple quadrupole time-of-flight mass spectrometer equipped with Analyst® TF 1.6 software (AB SCIEX, Foster City, CA). Both negative and positive mode was set for compounds ionization. The conditions of MS detector were as described elsewhere [8]. The chromatographic separation was carried out on a Shimadzu UFLC XR instrument (Shimadzu, Kyoto, Japan) and a Phenomenex Kinetex column (2.1 mm × 100 mm, 2.6 μm, Phenomenex, CA, USA). The mobile phase consisted of 0.1% aqueous formic acid (Sigma, St. Louis, USA, v/v, A) and acetonitrile (Sigma, St. Louis, USA, v/v, B) at a flow rate of 0.3 mL/min. The following gradient elution process were used: isocratic 2% solvent B (0–3 min), linear gradient from 2 to 60% solvent B (3–20 min), 60–100% solvent B (20–32 min), 2% solvent B for 8 min. The injection was 10 μL and the column temperature was 25 ℃.

The Ultimate 3000 DGLG System (Dionex, CA, United States) with DAD was applied for paeoniflorin determination. The mobile phase consisted of 0.1% aqueous formic acid (v/v) (A) and acetonitrile (B) run on a C18 Column (5 μm, 250 mm × 4.6 mm, Dionex CA, United States) at a flow rate of 1 mL/min. The following gradient elution process were used: isocratic 4% solvent B (0–10 min), linear gradient from 4 to 50% solvent B (3–30 min), 50–100% solvent B (30–35 min), 4% solvent B for 5 min. The DAD detector was set at 230 nm while the volume of injection was 10 μL.

### Data preparation and target prediction

Nineteen transition components of NSTC (9 prototype components and 10 metabolites) that had been detected in the brain in the previous study [8] were used for screening potential targets. Details of their chemical structures and information were shown in Additional file 1: Figure S1 and Table S1 respectively. The chemical structures of the 19 compounds were plotted using Chemical Draw 14.0 and saved in mol and sdf formats. Database retrieval, text information mining, reverse molecular docking and target prediction were performed to identify the putative target proteins of the NSTC comprehensively. We searched in TCMSP (https://lsp.nwu.edu.cn/tcmspsearch.php), TCMID (https://www.megabionet.org/tcmid/), Drugbank (https://www.drugbank.ca/) and other databases with the compound’s name as key words to collect the targets of the compounds. The online reverse molecule docking tool Pharmmapper (https://www.lilab-ecust.cn/pharmmapper/) was used to analyze the possible targets of small molecule compounds by submitting the sdf format file of the compound to the database. Target prediction was performed with the online target prediction tool TargetHunter, and the mol format files of compounds were imported into the online platform to find underlying targets of NSTC.

We searched stroke-related targets in TCMSP, TTD (https://db.idrblab.net/ttd/), DrugBank, OMIM (https://www.ncbi.nlm.nih.gov/omim) for related targets of stroke. Subsequently, text mining was also conducted to construct a database of stroke targets [11]. Wayne analysis was carried out to obtain the common targets of NSTC and stroke using software Venny (Version 2.1). The common targets were presumed to be putative targets that might exert pharmacological effects on stroke, and they would be used for later analysis.

### Network construction and analysis

We constructed a network of active components and putative targets of NSTC for the treatment of stroke based on their interactive data. The target proteins were analyzed using the mutiple-proteins model in the STRING database to construct the protein–protein interaction network (PPI). Cytoscape software (Version 3.0) was employed to visualize the PPI. Degree, betweenness centrality and closeness centrality were analyzed by the Network Analyzer plug-in in Cytoscape, which was used to measure the topological importance of nodes in the network. Target protein names were converted into the corresponding gene names by the Uniprot database and imported into the DAVID database for the KEGG pathway enrichment analysis.

### Animal experiments

One hundred and fifty-eight male C57BL/6 mice, weighed 18–25 g were provided by the Guangdong Medical Laboratory Animal Center (Guangzhou, China). The experimental protocol was approved by the Animal Ethics Committee of the School of Life Sciences at Sun Yat-sen University. All mice were housed in the animal room (25 ± 2 °C, 60 ± 5% relative humidity) with a 12 h dark/light cycle. The mice were assigned to five groups, the Sham group (n = 38), the ischemia stroke group (I/S group, n = 38), the NST 1 group (n = 22), the NST 2 group (n = 22), the NST 4 group (n = 38). Mice in the NST 1, 2, and 4 group were administrated with NSTC suspension at daily dose 0.47 (quivalent to the clinical dose, content powder/body weight), 0.94 (twice the clinical dose), and 1.87 g/kg (quadruple the clinical dose) once a day for 7 days. Mice in the Sham and I/S groups were administrated with pure water once a day for 7 days. The photochemical experiment was performed 30 min after the last administration on the 7th day.

### Experimental photothrombotic models

After the last administration on the 7th day, the animals were anesthetized with chloral hydrate (Aladdin Reagent, Shanghai China, 420 mg/kg). An incision was made in the middle of the animals’ scalp to expose and clean the skull. Within a 4 mm diameter region, the left parietal bone was polished to approximately 40–60 μm using a high-speed drill. The irradiation area center was 2 mm posterior to the bregma and 2 mm lateral to the sagittal suture. After the Rose Bengal dye (Bomei Biotechnology, Hefei, China, 100 mg/kg) was intraperitoneally injected, all the mice except for those in the Sham group were put on an electric blanket to maintain body temperature, and the mouse skull was illuminated with a 0.01 mW green laser for 15 min. The scalp was sutured, and the animals were allowed to recover. The mice were kept on administration once a day until sacrificed.

### Neurological function evaluation using mNSS

The mNSS (8 mice each group) was conducted at 24 h and on the 7th day after illumination to appraise neurological recovery of the stroke mice. It consisted of several aspects encompassing motor function, sensory, reflex, and balance tests, with full marks 18 points. The higher the score was, the more serious the nerve damage would be [12]. The detailed process of mNSS methodology and appraisal was shown in Additional file 1: Table S2. 30 min after mNSS test, the mice were sacrificed for western-blot and assay kit test.

### TUNEL assay and Nissl staining

Brains (3 mice each group) were collected from mice at 24 h and 7d after stroke which were perfused with 20 ml normal saline followed by 20 ml 4% paraformaldehyde, and were then fixed with 4% paraformaldehyde and dehydrated with 20% and 30% sucrose. The samples were embedded in OTC compound, frozen in liquid nitrogen, and sliced to a thickness of 20 μm using a freezing microtome (Leica CM1950, Leica, German). TUNEL staining and Nissl staining were carefully performed according to the manufacturers’ instructions by one-step TUNEL apoptosis detection kit (Beyontime Biotechnology, Shanghai, China) and Nissl Staining Solution (Beyontime Biotechnology, Shanghai, China) respectively. An anti-fluorescence quenching method was used during tissue sealing. The slides stained with TUNEL were observed under a fluorescence microscope (excitation wavelength ≈ 550 nm, emission wavelength ≈ 570 nm; Leica DM6B, German), and the ischemic area was photographed. The brain slices stained with Nissl were observed under a light microscope. Both Nissl- and TUNEL-positive cells in three randomly selected ischemic areas of the cerebral cortex were counted.

### RNA extraction and mRNA library construction

The mice for transcriptomics analysis (8 in each group) were sacrificed at 4 h, 24 h, and 7 d after surgery. Brains were obtained from the mice which were perfused with 20 ml physiological saline. The ischemic tissue of the mouse brain was quickly collected and stored in a centrifuge tube with RNAlater at − 80 °C for subsequent experiments.

Total RNAs were extracted from the samples using Trizol reagent (Invitrogen, Carlsbad, CA, USA) according to the manufacturer’s instructions. DNase I was used to digest double- and single-stranded DNAs in total RNAs. The cDNA was reversely transcribed from purified mRNA which was fragmented using buffer at an appropriate temperature. The PCR products were heat-denatured, and circularized by the oligo sequence. The single-strand circle DNA (ssCir DNA) was formatted as the final library. The final library was amplified and single-end 50 bases reads were generated on the BGISEQ500 platform (BGI, Shenzhen, China). Any unsatisfactory read obtained by sequencing was removed from the raw data to obtain clean reads. HISAT was used to compare the clean reads with the reference genome sequence. To determine the results whether meet the second quality control of alignment, the comparison rate and the distribution of reads on the reference sequence were compared. Quantitative gene analysis and cluster analysis based on gene expression levels were performed, and differentially expressed genes (DEGs) were screened out among the samples. The KEGG enrichment and GO enrichment data analysis was performed on the platform. According to the results of KEGG analysis, DEGs were functionally classified, and the phyper function in the R software was used for an enrichment analysis. The *p*-values of KEGG pathways and GO enrichment were calculated, and a *p* of < 0.05 was regarded as statistically significant.

### The detection of γ-aminobutyric acid (GABA) and Glutamic acid (Glu) level

GABA (Sigma, St. Louis, USA, 10 mg) and Glu (Sigma, St. Louis, USA, 10 mg) were dissolved by a mixed solution of formic acid, methanol and water (v:v:v = 2:200:800) to prepare a 1 mg/mL mixed standard stock solution. Then it was diluted to concentrations of 10,000, 8000, 6400, 3200, 1600, 800, 400, 200 ng/ml as working solutions respectively. Isoproterenol (25 mg) was dissolved in the same way to prepare a 2.5 mg/ml separate standard stock solution as an IS working solution. The brain tissue (50 mg) was homogenated with 1.89% formic acid solution (500 μL), centrifugated at 14,000 rpm, 4 °C for 10 min, and the supernatant was collected. 5000 μL 1.89% formic acid was added to 100 μL supernatant, and 90 μL mixture was transferred to a new EP tube, and then added with 10 μL working solution, 10 μL IS working solution. Afterward, 200 μL 1% formic acid-acetonitrile was added to obtain a mixture. It was vortexed, mixed, and centrifuged at 14,000 rpm for 10 min at 4 °C. The supernatant was collected for further analysis.

Samples (50 mg) were added with 180 μL 1.89% formic acid, homogenated, and centrifuged at 14,000 rpm for 10 min at 4 °C. Then the supernatant (2 μL) was mixed with 100 μL 1.89% formic acid, 10 μL IS working solution, and 200 μL 1% formic acid containing acetonitrile, and vortexed. The mixture was centrifuged at 14,000 rpm for 10 min at 4 °C. The supernatant was obtained for the analysis of GABA and Glu.

The analysis was performed on an Agilent 6410 Triple Quad LC–MS system (Agilent, USA). Chromatographic separation was carried out on an ACE 3 C18-PFP column (150 × 4.6 mm, 2.6 μm, A194318, UK) at 25 ℃. The mobile phase consisted of 0.1% formic acid (v/v) in both acetonitrile (A) and water (B) using a gradient-elution program, linear gradient 95% B at 0–2 min, 10% B at 2–5 min, 10% B at 5–6 min, and 95%B at 6–13 min. The injection volume was 10 μL with the flow rate kept at 0.6 mL/min.

The analytes and IS were ionized by the ESI source in positive ion mode and the ion spray voltage was set at 4000 V. The drying gas flow rate was 12.0 L/min; atomizing gas pressure was 30.0 psi with a source temperature of 325 °C. The Q1 Mass (Da)/Q3 Mass (Da) of Glu, was 148.0/84.0 while GABA was 104.0/45.1. The fragmentation voltage of Glu and GABA was 75 V and 65 V, with collision voltage 13 V and 22 V respectively.

### Western blotting analysis

The protein levels of Bax, Bcl-2, and *N*-methyl-d-aspartate receptor (NMDAR) 1 in brain tissue were quantitated using western blotting analysis. Brain tissues from left hemisphere lysed with 19-fold-volume, ice-cold RIPA lysis buffer were homogenated with on the ice, and centrifuged at 10,000 rpm. The supernatant was obtained for further experiments. Total protein concentration was determined by the enhanced BCA protein assay (Beyondtime Biotechnology, Shanghai China). The protein samples were loaded onto a 10% resolving SDS-PAGE gel and a 10% stacking gel, and fractionated by electrophoresis at 250 V. They were electrotransferred onto PVDF membranes which were subsequently washed three times with Tris-buffered saline containing 0.1% Tween-20 (TBST) for 5 min each time, and blocked in 5% non-fat dry milk in TBST for 2 h. Afterward, they were incubated with primary antibodies, encompassing anti-Bax, anti-Bcl-2 and anti-NMDAR1 (diluted 1:1000, Abam Technology, Cambridge, UK), overnight at 4 °C, and washed three times. The membranes were incubated with the secondary antibody (diluted 1:5000) for 1 h. Protein bands were visualized using a chemiluminescence (ECL) assay kit and photographed using a Syngene Tanon5200 imaging system (Tanon, China). Besides, *β*-actin (diluted 1:10,000, Santa Cruz Dallas, TX, USA) was used as an internal reference. Expression levels of all proteins were normalized to that of *β*-actin. The optical density of the bands was determined using Image J software.

### SOD, GSH, IL-1*β,* TNF-*α* and TGF-*β*1 assay of Brain tissues biochemistry analysis

After neurological evaluation was completed, the left hemisphere of the brain was completely moved out and stored at – 80 ℃. Brain tissues (30 mg) from left hemisphere were added with pre-chilled cell lysates (600 μL), homogenated on ice, and centrifugated at 3500 rpm. The supernatant was collected. The protein concentration was determined using a BCA assay. The SOD activity, GSH content at were tested by assay kits (Nanjing Jiancheng, Nanjing, China) and expressions The ELISA ssay kits of IL-1*β* (Meimian, Wuhan, China), TNF-*α* (Meimian, Wuhan, China) and TGF-*β*1 (Cusabio, Wuhan, China) were used to quantitate the expression level according to the manufacturers’ instructions.

### Data analysis

All data were analyzed using Graphpad prismprogram 5.0 and presented as means ± SEM. One-way ANOVA (nonparametric test) with Dunnett post-hoc test was used to analyzed the histopathological data. The other data was analyzed by one-way ANOVA with Bonferroni post-hoc test. A *p* value < 0.05 was considered statistically significant.

## Result

### Main compounds of NSTC

**Fig. 1:**
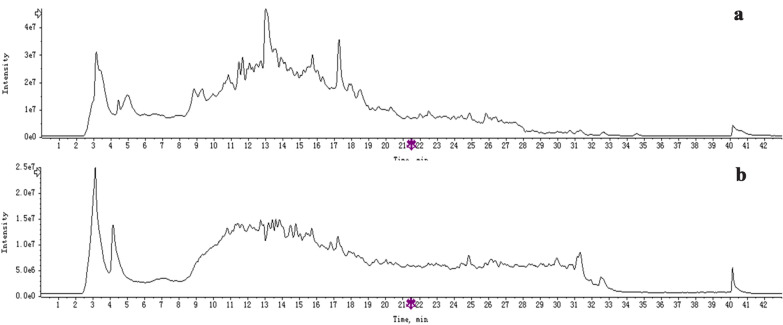
**a** TIC of NSTC in negative mode. **b** TIC of NSTC in positive mode

By comparing the retention time and MS^n^ spectrum with reference standards, 10 compounds were confirmed, and other 32 constituents of NSTC were identified according to their MS^n^ spectrum. The detailed characteristics of the compounds were summarized in Additional file 1: Table S3. The total ion chromatograms (TIC) of both positive and negative modes were shown in Fig. 1.

### The content of paeoniflorin in NSTC

Based on an established HPLC method, good linearity of paeoniflorin was achieved with a correlation coefficient of R^2^ = 0.9997, linear equation y = 0.4695x−0.0081. The chromatograms of paeoniflorin and NSTC were shown in Additional file 1: Figure S2. The content of paeniflorin was 40.70 mg/g.

### NSTC rescued neurological deficits and curbed ischemic damages

**Fig. 2:**
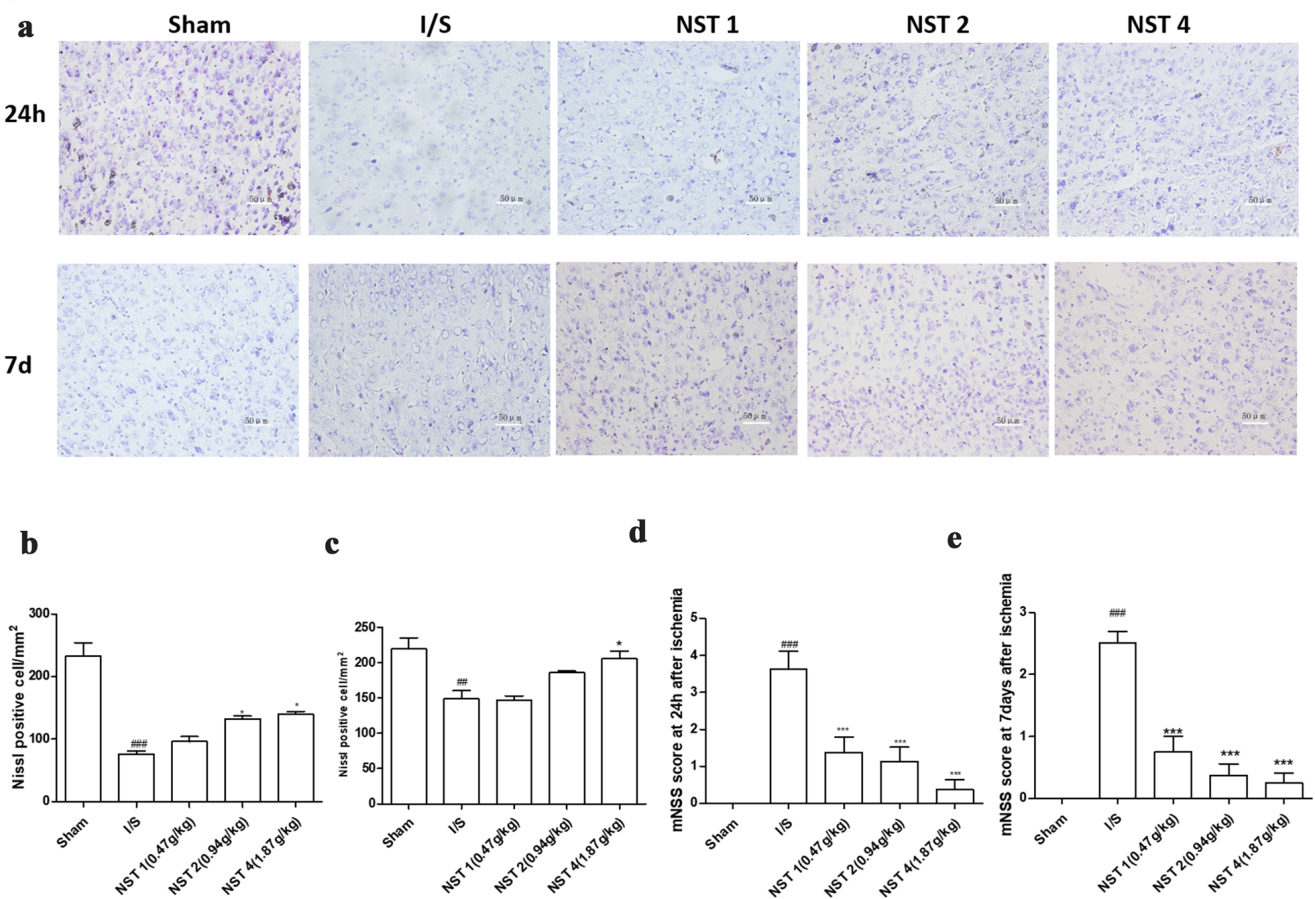
**a** Light microphotographs of the brain tissue with Nissl staining (scale bar = 50 μm). **b** The effects of NSTC on increasing Nissl positive cell at 24 h after ischemia stroke, n = 3, *F* = 30.48, *p* < 0.0001. **c** The effects of NSTC on increasing Nissl positive cell at 7 days after ischemia stroke, n = 3, *F* = 9.756, *p* = 0.0018 **d** mNSS score at 24 h after stroke, n = 8, *F* = 15.32, *p* < 0.0001. **e** mNSS score at 7 days after stroke, n = 8, *F* = 31.65, *p* < 0.0001. The data were One-way ANOVA with Bonferroni post-hoc test, expressed as means ± SEM, #*p* < 0.05, ^##^*p* < 0.01, ^###^*p* < 0.001 vs the Sham group, ^*^p < 0.05, ^**^p < 0.01, ^***^p < 0.001 vs the I/S group

The severity of neurological injury after stroke was evaluated using Nissl staining and mNSS. The results showed that different degrees of neurological damages occurred in all except the Sham group after 24 h and 7 d of modeling. The majority of cells in the Sham group had clear borders and Nissl bodies at 24 h, with the density of surviving neurons of 232.63 ± 37.38 cells/mm^2^. Compared with the Sham group, the Nissl-positive cell count and the density of surviving neurons (76.49 ± 8.04 cells/mm^2^) in the IS group significantly decreased at 24 h (*p* < 0.001). Higher densities of Nissl-positive cells were observed in the NST 2 (132.28 ± 8.44 cells/mm^2^ vs. 76.49 ± 8.04 cells/mm^2^), *p* < 0.01) and 4 groups (139.65 ± 7.97 cells/mm^2^ vs. 76.49 ± 8.04 cells/mm^2^), *p* < 0.001). After 7 d of stroke modeling, the density of Nissl-positive cells significantly decreased in the IS group (148.77 ± 19.93 cells/mm^2^) compared with the Sham group (219.30 ± 27.48 cells/mm^2^, *p* < 0.05). Compared with the IS group, the density of surviving neurons significantly increased in the NST 2 (185.62 ± 4.38 vs. 148.77 ± 19.93 cells/mm^2^, *p* < 0.05) and 4 groups (205.26 ± 18.95 cells/mm^2^ vs. 148.77 ± 19.93 cells/mm^2^, *p* < 0.05) (Fig. 2a–c), and the mNSS scores significantly decreased in all NSTC groups (Fig. 2d, e). These results indicated that NSTC rescued neurological damages induced by stroke.

### Putative targets of NSTC for the treatment of stroke

**Fig. 3:**
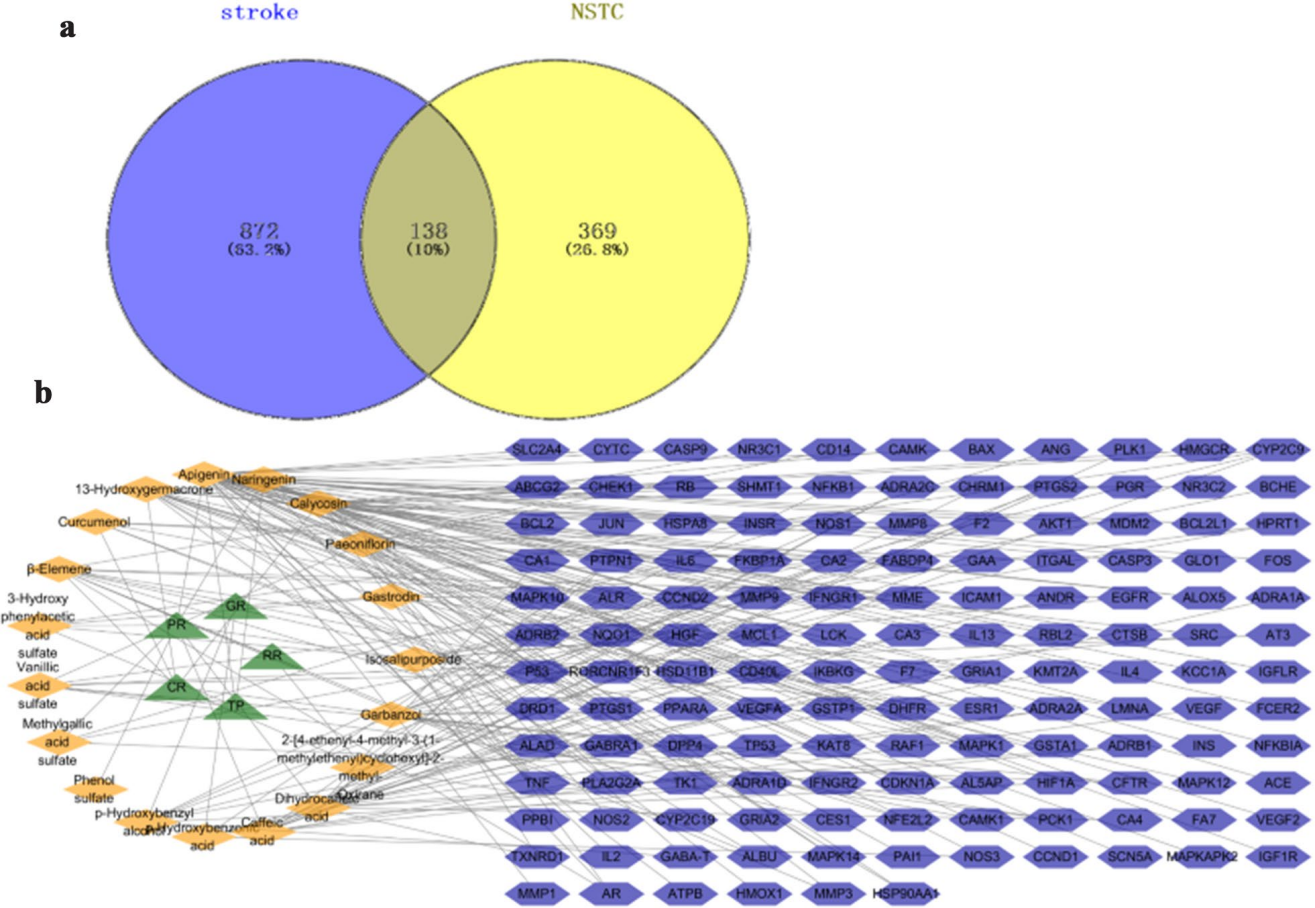
Network construction and analysis **a** Venn diagram of candidate targets in NSTC and stroke. **b** The Component-Target network. The green circles refer to 5 herbs composed of NSTC. The red diamonds represent prototype components and metabolites of NSTC in brain. The blue hexagons represent putative targets of NSTC for the treatment

A total of 507 potential therapeutic targets of active components in NSTC were predicted based on database retrieval, text information mining [13–15], reverse molecular docking, and target predictions, while 1010 stroke-related targets were obtained based on database retrieval and text information mining. In comparisons with the two groups of targets, there were 138 overlapping targets, as shown in Fig. 3a. We presumed those targets as the putative targets of NSTC for the treatment of stroke and constructed the herb-compound-target network (Fig. 3b). Apigenin associated with 50 targets that exerted therapeutic effects on stroke, followed by calycosin which had 24 stroke-related targets and 13-hydroxygermacrone that owned 16 targets.

### Compound-target network analysis

The data of therapeutic targets of NSTC against stroke was imported to the DAVID database for the KEGG pathway enrichment analysis (*p* value < 0.05), and 116 pathways were enriched.

Data were also submitted to the STRING database to establish PPI. The PPI was introduced into Cytoscape 3.1, with target proteins as nodes and interactions as edges. The analysis was carried out considering the three parameters, degree, betweenness, and center closeness. The results were shown in Additional file 1: Table S4. There were 79 nodes with degree > 10, indicating that the proteins of each target were closely related.

### The DEGs affected by NSTC

In the present study, a total of 18,928 genes were detected. Compared with the Sham group, 95, 184 and 156 genes were up-regulated in the I/S group and 101, 34 and 127 genes were down-regulated after 4 h, 24 h and 7 d of modeling. Compared with the I/S group, 333, 240 and 121 genes were up-regulated in the NST 4 group and 463, 195 and 79 were down-regulated in the NST 4 group after 4 h, 24 h, and 7 d of modeling, respectively.

**Fig. 4:**
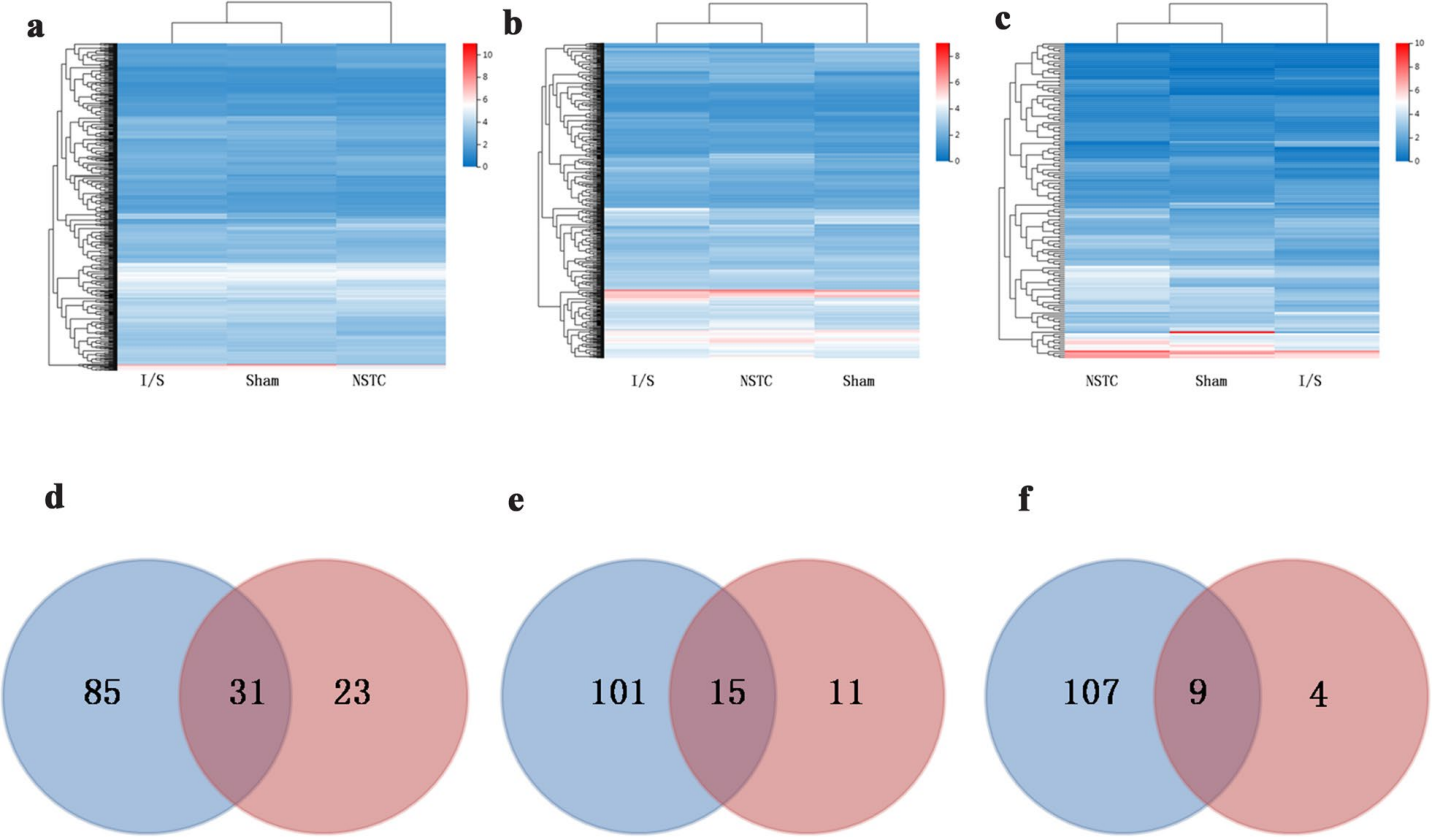
Cluster analysis of gene expression of different groups at 4 h (**a**), 24 h (**b**), 7d (**c**) after ischemia. The more the color is red, the higher the expression, the more the color is blue, which means the lower the expression. The common signaling pathways shared by 138 putative targets and DEGs at 4 h (**d**), 24 h (**e**), 7d (**f**) after ischemia stroke. The blue circle represented the number of pathway enriched by 138 putative targets, the red circle represented the DEGs

Gene expression levels of the samples were clustered separately according to the ischemic time. The results were presented in a visual heat map (Fig. 4a–c), and the longer the topological distance was, the greater the differences between gene expressions would be. After 4 h of modeling, the topological distance between the I/S and NSTC groups increased, indicating that NSTC exerted therapeutic effects at the early stage (Fig. 4a). After 24 h of stroke modeling, the the topological distance between the I/S and Sham groups increased, which meant that gene expressions significantly changed at 24 h. This indicated that NSTC had a certain callback effect on gene expressions (Fig. 4b). After 7 d of stroke modeling, the NSTC and Sham groups had similar levels of gene expressions, indicating that NSTC facilitated the recovery from stroke (Fig. 4c).

### The KEGG enrichment of DEGs

The KEGG-enriched pathways of DEGs were screened with a *p* value threshold of < 0.05. After 4 h of stroke modeling, 17 signaling pathways were significantly enriched between the Sham and I/S groups, incorporating inflammation-related pathways, coagulation- and metabolism-related pathways, thrombosis-related pathways, neurotransmitter-involved pathways and synapse-associated pathways, which were consistent with clinical symptoms of stroke. Fifty-four signaling pathways were enriched for DEGs between the NSTC and IS groups, covering signal transductions, inflammatory responses, neurotransmitters and synapses, as well as cell apoptosis. These results suggested that NSTC started to exert therapeutic effects within 4 h after stroke, and the main mechanism that the TCM formula repressed acute inflammatory responses, curbed cell apoptosis in neurons, restored signal transmission and regulated neurotransmitters synapses.

Thirty-five signaling pathways were enriched between the Sham and IS groups at 24 h, which were mainly related to inflammatory responses. Moreover, 26 signal pathways were enriched for the DEGs between the NSTC and I/S groups, which were mainly related to inflammatory responses, neurotransmitters synapses that involved in signal transmissions, and other signaling pathways.

Seven signaling pathways that were involved in neurotransmitters and synapses, metabolism and the cell cycle were enriched for the DEGs between the Sham and I/S groups after 7 d of stroke modeling. Thirteen signaling pathways associated with cell cycle, inflammatory responses, asignal-transmission and neurotransmitters synapses were enriched for the DEGs between the NSTC and I/S groups.

Wayne analysis was carried out to confirm the common pathways based on results of network pharmacology and transcriptomics analysis. It was found that there was 31 (Fig. 4d), 15 (Fig. 4e), and 9 (Fig. 4f) common signaling pathways shared by 138 putative targets and DEGs at 4 h, 24 h and 7 d, respectively. These signaling pathways were listed in Additional file 1: Table S5.

### The GO enrichment of DEGs

The major GO terms of DEGs between the IS and Sham group at 4 h were regulations of multicellular organismal process and development. Similar trends also existed between the NSTC and I/S groups. The major GO terms of DEGs between the I/S and Sham groups at 24 h were related to immune activities, indicating that immune-inflammatory genes played predominant roles in stroke at 24 h and that NSTC significantly suppressed inflammatory responses. The DEGs between the NSTC and I/S groups were enriched in terms of multicellular organisimal development and tissue development on the 7th day of modeling, indicating that NSTC promoted ischemic brain tissue repair.

### NSTC suppressed inflammatory and anti-oxidant activities and up-regulated cerebral TGF-*β*1 expressions

**Fig. 5:**
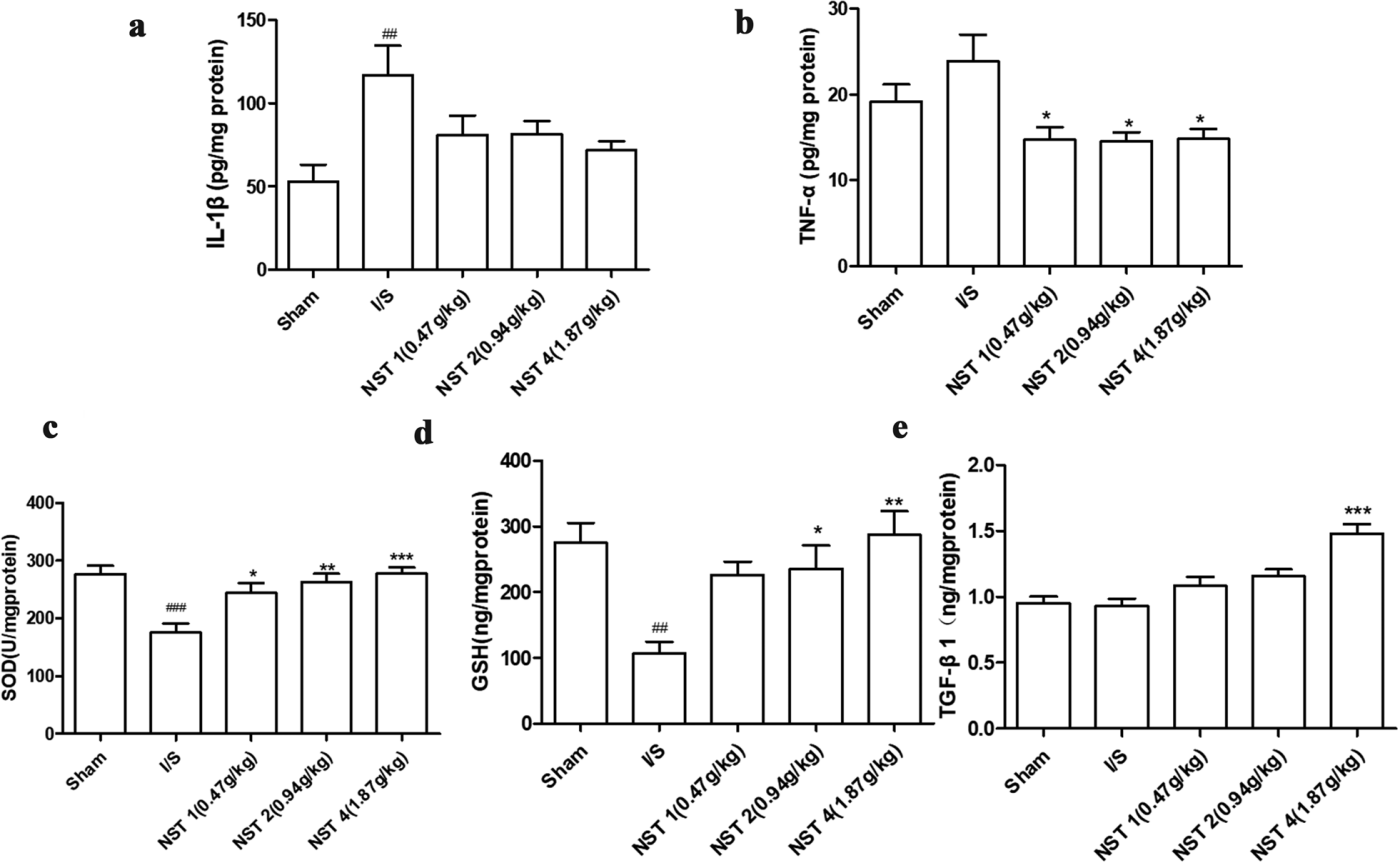
Effects of NSTC on the expression of TNF-α (**a**, *F* = 4.464, *p* = 0.0051), IL-1β (**b**, *F* = 4.270, *p* = 0.0064), the activity of SOD (**c**, *F* = 8.001, *p* = 0.0001), the content of GSH (**d**, *F* = 6.151, *p* = 0.0007) and TGF-β1 (**e**, *F* = 14.01,* p* < 0.0001). The data were analyzed by one-way ANOVA with Bonferroni post-hoc test, expressed as means ± SEM, n = 8, ^#^*p* < 0.05, ^##^*p* < 0.01, ^###^*p* < 0.001 vs the Sham group, ^*^p < 0.05, ^**^p < 0.01, ^***^p < 0.001 vs the I/S group

IL-1*β* and TNF-*α* were two important inflammation indicators. The results showed that compared with the Sham group, expression levels of IL-1*β* and TNF-*α* in the I/S group increased at 24 h. This indicated that the inflammatory responses occurred in the brain after photochemical stroke. Compared with the I/S group, cerebral IL-1*β* and TNF-*α* levels decreased in each NSTC group (Fig. 5a, b).

SOD and GSH were two important oxygen free radical scavengers in humans, reflecting the regulatory ability to scavenge oxygen free radicals. Compared with the I/S group, the activity of SOD in the NST 2 and 4 groups significantly increased, and so did the GSH content in all NST groups at 24 h after stroke. (Fig. 5c, d).

TGF-*β*1 is considered to be critical in brain tissue repair. As the 7th day after stroke was a crucial time point for rehabilitation for stroke, TGF-*β*1 levels were particularly observed on the very day. It was found that TGF-*β*1 expressions in the NST 4 groups significantly increased compared with the I/S group (Fig. 5e), which confirmed the therapeutic efficacy of NSTC in brain tissue repair after ischemic stroke.

### NSTC curbed cell apoptosis in ischemic neurons

**Fig. 6:**
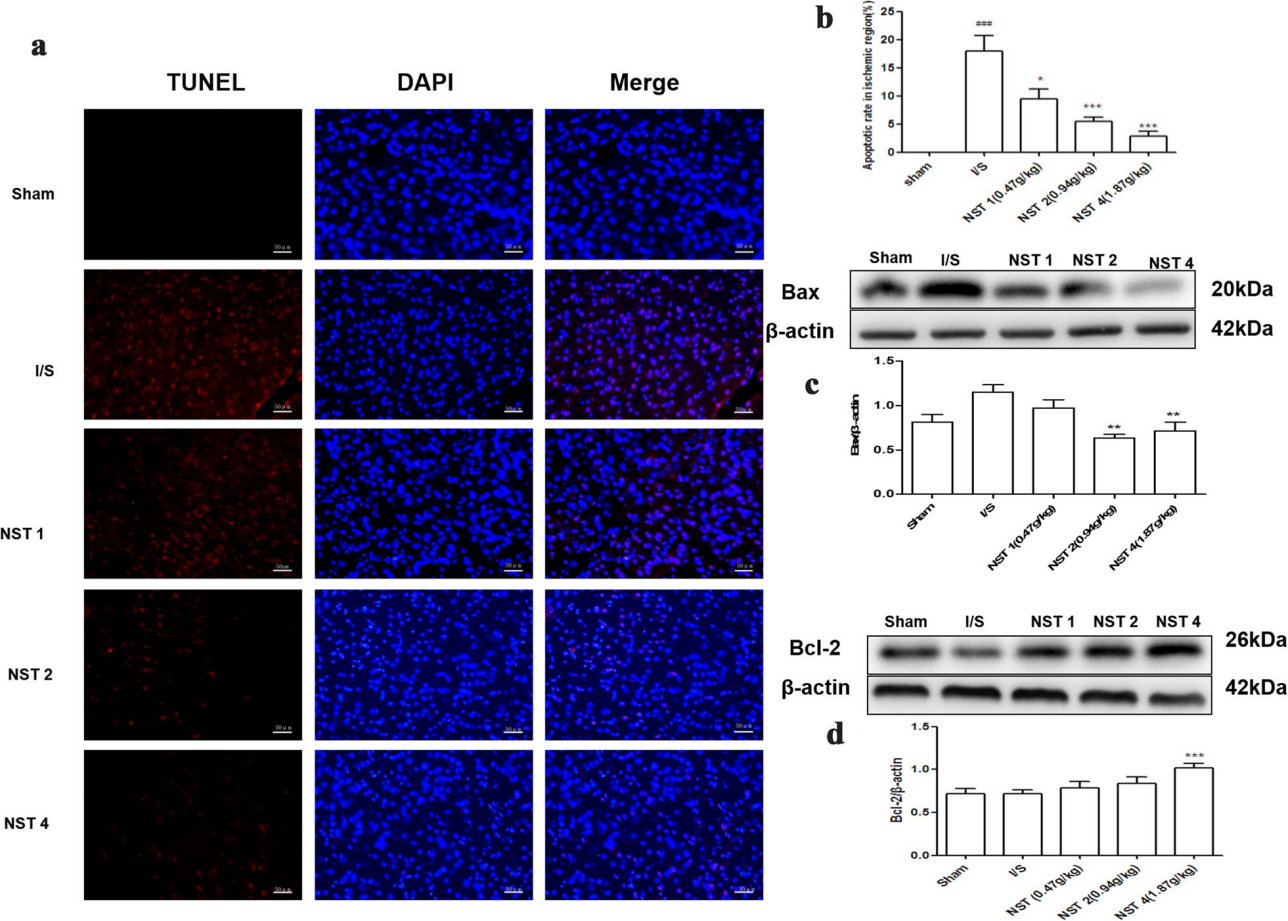
**a** The fluorescence microphotographs of TUNEL analysis (scale bar = 50 μm). **b** The effects of NSTC on apoptosis rate, n = 3, *F* = 18.96, *p* = 0.0001. **c**, **d** The effects of NSTC on regulating the expressions of Bax *(F* = 6.072, *p* = 0.0008) and Bcl-2 (*F* = 3.932, *p* = 0.0097), n = 8. The data were analyzed by One-way ANOVA with Bonferroni post-hoc test, expressed as means ± SEM, ^#^*p* < 0.05, ^##^*p* < 0.01, ^###^*p* < 0.001 vs the Sham group, ^*^p < 0.05, ^**^p < 0.01, ^***^p < 0.001 vs the I/S group

Stroke often brought about cell apoptosis in ischemic neurons and TUNEL staining was a commonly used method for detecting cell apoptosis. The results revealed cell apoptosis in the ischemic area at 24 h. As shown in Fig. 6a, b, the apoptosis rates significantly decreased in all NSTC groups. Meanwhile, one of the apoptosis-related proteins Bax (Fig. 6c) was significantly up-regulated, while another apoptosis-related protein Bcl-2 (Fig. 6d) was significantly down-regulated in the NSTC groups.

### NSTC reduced the toxicity of EAAs

**Fig. 7:**
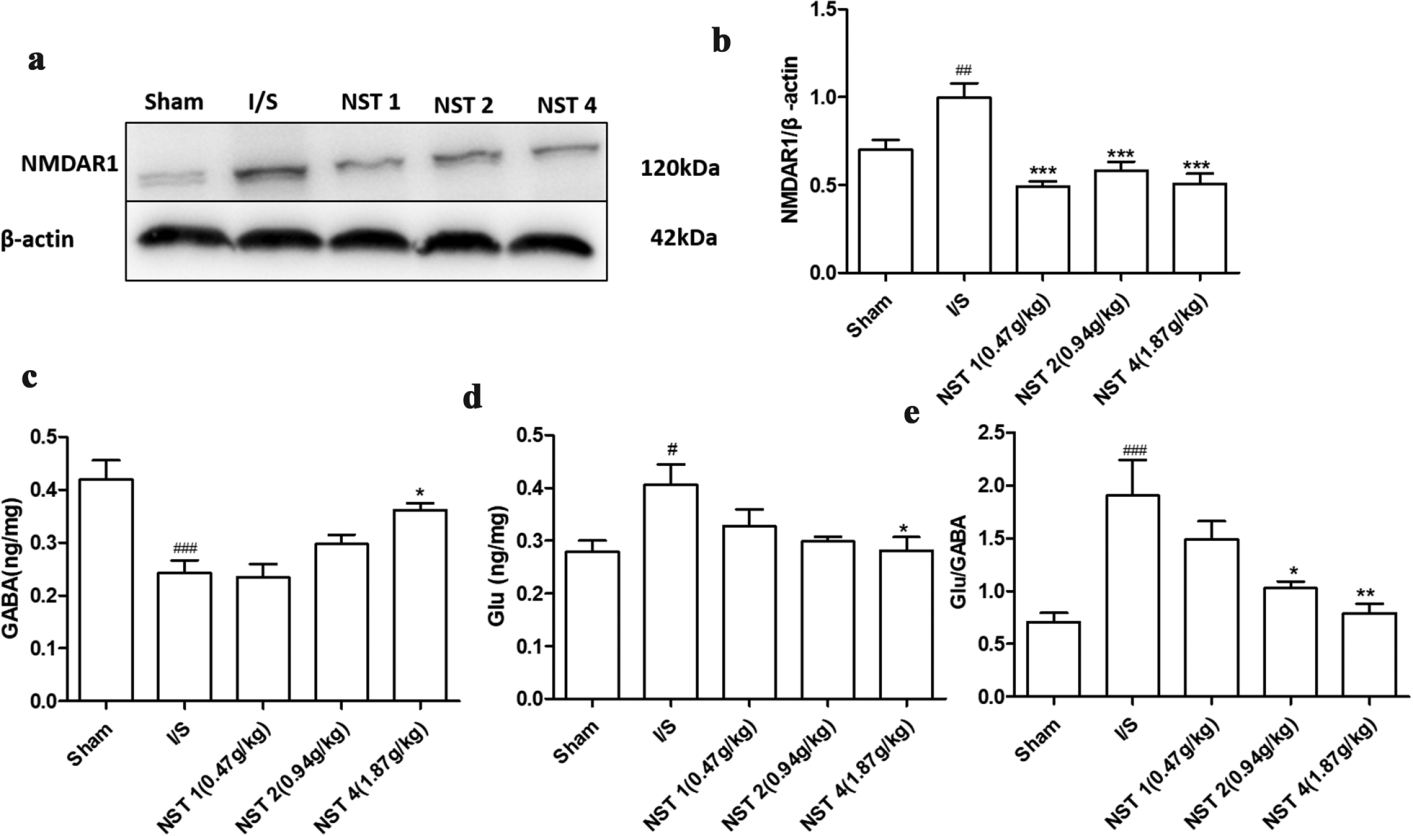
**a**, **b** The effects of NSTC on the expression of NMDAR1, n = 8, *F* = 12.78, *p* < 0.0001. **c** The effects of NSTC on the content of GABA, n = 8, *F* = 10.31, *p* < 0.0001. **d** The effects of NSTC on the content of Glu, n = 8, *F* = 3.745, *p* = 0.016. **e** The effects of NSCT on the value of Glu/GABA, n-8, *F* = 7.734, *p* = 0.0001. The data were analyzed by One-way ANOVA with Bonferroni post-hoc test, expressed as means ± SEM, n = 8,^#^*p* < 0.05, ^##^*p* < 0.01, ^###^*p* < 0.001 vs the Sham group, ^*^p < 0.05, ^**^p < 0.01, ^***^p < 0.001 vs the I/S group

Levels of NMDAR1, Glu and GABA, as well as the Glu/GABA ratio were main indicators to evaluate the toxicity of EAAs. The results showed that expressions of NMDAR1 and Glu, and the Glu/GABA ratio significantly increased at 24 h, meanwhile the GABA expression decreased in the I/S group compared with the Sham group. In the NSTC groups, cerebral expressions of NMDAR1 and Glu and the Glu/GABA ratio decreased compared with the I/S group. Meanwhile, the GABA expression was up-regulated with NSTC administration (Fig. 7).

## Discussion

A decrease in cerebral blood flow induced by cerebral arteriosclerosis, thrombosis, and vasospasm is the main pathogenic factor of ischemic stroke. The progression of stroke is often divided into the superacute phase (0–6 h), acute phase (6–24 h) and subacute phase (1d–14d) [16]. As NSTC has been widely used as a TCM prescription for stroke in the clinic, its efficacy in acute- and subacute-phase stroke is gradually being accepted and corroborated by growing clinical evidences. In this study, the results of Nissl staining and mNSS have shown that NSTC ameliorates neurological function and reduces neuronal damages in stroke mice. The heat map of relevant gene expressions in the superactue phase (4 h), acute phase (24 h), and subacute phase (7 d) also confirms that NSTC is beneficial to the treatment and rehabilitation of stroke. These results are consistent with other clinical trials and previous pharmacological studies on NSTC. Moreover, our results also suggest that the pretreatment of NSTC aids in ameliorating the outcome of ischemic stroke mice. Therefore, it sheds new light on the clinic application of NSTC, which probably can be used for the prevention of stroke in high-risk patients.

Network pharmacology is regarded as a powerful tool for identifying multi-components and investigating mechanisms of therapeutic efficacy of Chinese herbal medicines [17]. Selecting ingredients for screening is a critical step for further network pharmacology research. At present, most ingredients selected for screening are based on database retrieval or virtual computing which can not yet completely reflect the actual absorption, metabolism, and distribution of TCM in vivo. Modern pharmacological evidences confirm that bioactive ingredients of drugs reaching the target organs is the vital prerequisite for their effectiveness. Based on a previous study [8], 19 transitional components detected in the brain have been selected as active ingredients for target predictions.

Among the 19 transition components, apigenin has the most targets (50 targets) and its corresponding targets are at the center of the PPI network. It indicates that apigenin is one of the core components of NSTC to exert anti-ischemic stroke effects. According to the previous study [8], apigenin is mainly generated by typhaneoside from TP and originated from PR. Apigenin may be the active form of typhaneoside in vivo. Previous studies have confirmed that apigenin has therapeutic effects on stroke—for example, apigenin showed protective effects on acute focal cerebral ischemia/reperfusion injury [18] and permanent cerebral ischemia [19]. In addition to apigenin, the herbs-active components-targets network, PPI network and results of KEGG analysis also reveal that the other components, such as paeoniflorin in PR, calycosin in CR, naringenin in TP, *β*-elemene in CR and RR, gastrodin in GR, 13-hydroxygemacone in CR, garnzol in CR, caffeic acid in PR, and *p*-hydroxybenzyl alcohol in GR, also contribute to the pharmacological effects of NSTC. The results demonstrate that the therapeutic efficacy of NSTC is a result of multi-component, multi-target, and multi-path synergies.

As the supply of oxygen and glucose is interrupted due to cerebrovascular embolization-induced ischemia, a series of cascade reactions mainly including inflammatory responses, EAAs toxicity and free radical release ultimately lead to cell apoptosis in ischemic neurons and resultant brain tissue damages [20]. Rescuing ischemic neurons in the acute phase is crucial for the treatment of acute ischemic stroke. Anti-thrombolytic drugs are mainly used for reversible ischemic tissues by dissolving thrombus, improving cerebral circulation and recanalizing occluded arteries within the therapeutic time window. However, limited by the short time window (< 4.5 h) and medical condition, many patients can not receive timely treatment. Therefore, neuroprotective drugs become a hot spot in stroke research. Anti-inflammation, alleviating the toxicity of EAAs, anti-oxidant activity, anti-apoptosis in damaged neurons are 4 main goals of neuroprotective drugs for acute-phase stroke.

Immune-inflammation is predominant in the pathophysiology of ischemic stroke, especially acute-stage ischemic stroke. The aggravation of cerebral inflammation increases intracranial pressure, damages the blood–brain barrier, causes brain edema, and even leads to death [21]. In our study, the two important inflammatory biomarkers, IL-1*β* and TNF-*α*, significantly decrease in the NSTC groups. The transcriptomics analysis shows that NSTC suppresses activities of inflammation-related pathways in the superacute and acute phases, including the TNF-*α* signaling pathway that exacerbates immune-inflammatory responses and triggers cell apoptosis in damaged neurons by binding to specific receptors on the cell membrane [22], the Toll-like receptor signaling pathway that plays an important role in ischemic stroke [23], the NOD-like receptor signaling pathway, etc. In addition, NSTC also suppresses the activity of the PI3K-Akt signaling pathway, the NF-*κ*B signaling pathway, the MAPK signaling pathway and other important signal transduction pathways initiated by inflammation. The network showed that the apigenin-related targets included AKT1, TNF, and MAPK1. It is speculated that apigenin may lower expressions of inflammatory factors and inhibit inflammatory responses via regulating the PI3K-Akt, T cell receptor, Toll-like receptor, and NOD-like receptor signaling pathways after the interactions with AKT1, TNF, and MAPK1. Other components presenting anti-inflammatory effects are as follows: naringenin targets to TNF and MAPK1, gastrodin to MAPK12, *β*-elemene to MAPK1, calycosin to MAPK14, which thereby inhibit activities of the TNF signaling pathway, the MAPK signaling pathway, and the T cell receptor signaling pathway synergetically. Paeoniflorin targets to the pro-inflammatory cytokine IL6. Caffeic acid, a metabolite of chlorogenic acid in PR, and garnzol, a metabolite of calycosin, both target to PTGS2 which is an important target of the NF-*κ*B signaling pathway [24] triggered by inflammation. All these components can suppress immune-inflammatory responses synergetically.

Extracellular accumulation of EAAs during ischemic stroke can excite NMDAR1 and resultant Ca^2+^ influx, which further destroy the mitochondrial membrane potential, leading to the damage of mitochondrial, decreases in ATP synthesis, dysfunction of Na^+^/K^+^-ATPase, and finally cell apoptosis in ischemic neurons [25, 26]. Glu as the most important EAAs mainly binds to and excites NMDAR1. GABA receptors as the main inhibitory amino acid receptors activated by GABA can antagonize EAA toxicity caused by Glu, exerting neuroprotective effects [27]. The herb-component-target network showed that 13-hydroxygemacone, gastrodin and its metabolite *p*-hydroxybenzyl alcohol target to *GRIA1* (encoding NMDAR1). Furthermore, gastrodin is able to down-regulate GABA-T expressions [10] and elevate GABA levels to alleviate excitatory amino acid toxicity. Our pharmacological experiments have shown that NSTC down-regulates the protein expression of NMDAR1. Meanwhile, GABA expressions significantly increase after NSTC administration, along with decreased Glu levels and the Glu/GABA ratio. In a word, 13-hydroxygemone, gastrodin, and *p*-hydroxybenzyl alcohol present antagonistic effects against excitatory amino acid toxicity in acute-phase ischemic stroke.

SOD and GSH-Px are two non-mitochondria enzymes in vitro and important endogenous oxygen free radical scavengers. AKT1, one of the apigenin-related targets, is able to increase SOD and GSH-Px activities, scavenging oxygen free radicals in focal cerebral ischemia [28]. NSTC has been proven to enhance the removal of oxygen free radicals and apigenin may be the biggest contributor. NSTC also shows anti-apoptotic activity against stroke by down regulating the apoptosis signaling pathway and Bax levels, up-regulating Bcl-2 expressions. Moreover, the two apoptosis-related proteins are also the targets of apigenin. The network pharmacology results suggest that apigenin is related to the apoptosis signaling pathway and anti-apoptosis can be another mechanism of apigenin behind the anti-stroke effects of NSTC.

Subacute-phase intervention is a crucial factor affecting the quality of recovery with minimum sequelae after stroke. Our results have shown that NSTC up-regulates cerebral TGF-*β*1 expressions on the 7th day of stroke. TGF-*β*1 is reported to promote brain tissue repair after stroke and regulate the differentiation of microglial cells [29]. Our results suggest that the MAPK signaling pathway-related components, apigenin, naringenin, calycosin, gastrodin, *β*-elemene, may be the active ingredients of NSTC to promote brain tissue repair after stroke.

**Fig. 8:**
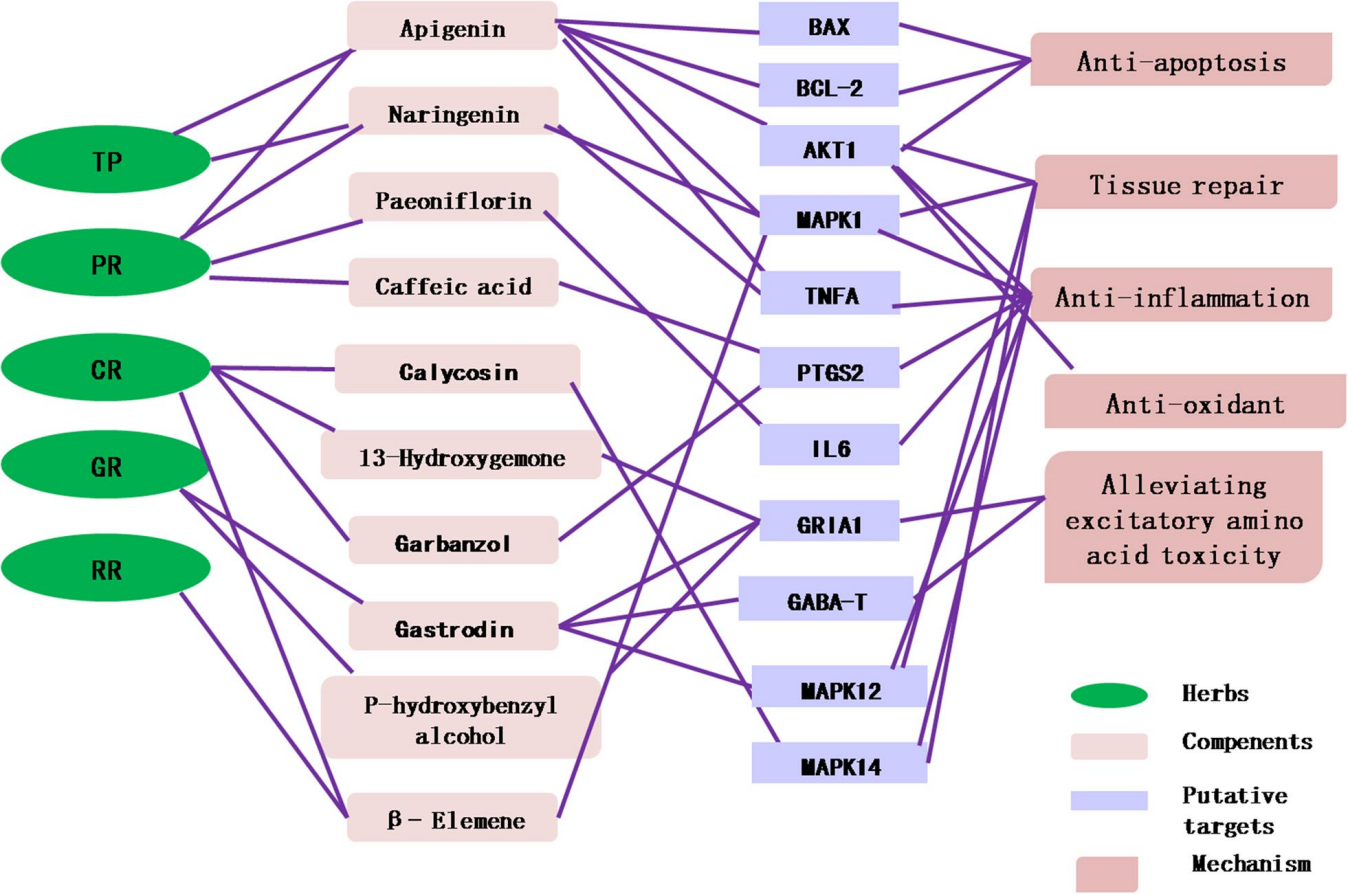
The holistic mechanism of NSTC in the treatment of stroke

In summary, the present study demonstrates that NSTC exerts potent and preventive neuroprotective effects on ischemic stroke by inhibiting immune-inflammatory responses, anti-oxidant activities and cell apoptosis in ischemic neurons, as well as curbing EAA toxicity. Furthermore, NSTC promotes brain tissue repair in the subacute stage of stroke. Apigenin as the most active ingredient in NSTC shows robust anti-inflammatory, anti-apoptotic, anti-oxidant and tissue-repair effects. Besides, other components also contribute to the neuroprotective effects of NSTC. The possible underlying mechanism and pharmacological substances have been listed in Fig. 8. The synergy of various components exactly reflects the holistic view of TCM.

## Conclusion

Stroke has been a publish health concern and a social problem in the world. While TCM has been proven to have unique advantages in the treatment and rehabilitation of stroke, the elucidation of the pharmacological mechanism and substance basis are becoming emerging bottlenecks restricting the research and development of TCM. The present study identifies the active ingredients of NSTC and illustrates the underlying mechanism using a combination of network pharmacology, transcriptomics analysis, and pharmacological experiments. Our results may inspire more studies to clarify precise molecular mechanisms behind the effects of these active ingredients on ischemic stroke.

## Supplementary information

**Additional file 1: Table S1.** The absorbed constituents and metabolites detected in brain. **Figure S1.** The absorbed constituents and metabolites detected in brain. **Table S2.** Test terms and scoring standards of mNSS. **Table S3.** Identification of phytochemical components in NSTC and absorbed components in mice tissues. **Table S4.** Topology characteristics of hub nodes from PPI network. **Table S5.** The common signaling pathways shared by 138 putative targets and differentially expressed genes at 4h, 24h, 7d after ischemic stroke.

## Data Availability

All data generated or analyzed during this study are included in this published article.

## Abbreviations

NSTC: Naoshuantong capsule
TP: Typhae pollen
PR: Radix Paeoniae Rubra
CR: Curcumae Radix
GR: Gastrodiae Rhizoma
RR: Radix Rhapontici
TCM: Traditional Chinese medicine
mNSS: Modified neurological severity scores
EAAs: Excitatory amino acids
CFDA: China Food and Drug Administration
MS: Mass spectrometry
PPI: Protein–protein interaction network
I/S group: The ischemia stroke group
DEGs: Differentially expressed genes
GABA: γ-Aminobutyric acid
Glu: Glutamic acid
IS: Isoproterenol
NMDAR: *N*-Methyl-d-aspartate receptor
TBST: Tris-buffered saline containing 0.1% Tween-20
ECL: Chemiluminescence
TIC: Total ion chromatograms
