# Supplementary material for: The study of neuroprotective effects and underlying mechanism of Naoshuantong capsule on ischemia stroke mice
Source: Chin Med. 2020 Nov 17;15:119. doi: 10.1186/s13020-020-00399-7 (PMC7670690; doi:10.1186/s13020-020-00399-7)
Supplement: Supplementary file 1 — Additional file 1: Table S1. The absorbed constituents and metabolites detected in brain. Figure S1. The absorbed constituents and metabolites detected in brain. Table S2. Test terms and scoring standards of mNSS. Table S3. Identification of phytochemical components in NSTC and absorbed components in mice tissues. Table S4. Topology characteristics of hub nodes from PPI network. Table S5. The common signaling pathways shared by 138 putative targets and differentially expressed genes at 4h, 24h, 7d after ischemic stroke. [file 13020_2020_399_MOESM1_ESM.doc]

Table 1 The absorbed constituents and metabolites detected in brain

|  | compound | herb |
| --- | --- | --- |
| Prototype constituents | Apigenin | TP, PR |
|  | Naringenin | TP |
|  | 13-Hydroxygermacrone | CR |
|  | Curcumenol | CR |
|  | β-Elemene | CR，RR |
|  | Calycosin | CR |
|  | Paeoniflorin | PR |
|  | Isosalipurposide | PR |
|  | Gastrodin | GR |
| metabolites | Vanillic acid sulfate | PR, TP |
|  | Methylgallic acid sulfate | PR |
|  | Phenol sulfate | PR |
|  | p-Hydroxybenzyl alcohol | GR |
|  | p-Hydroxybenzonic acid | GR |
|  | Caffeic acid | PR |
|  | Dihydrocaffeic acid | PR |
|  | Garbanzol | CR |
|  | 3-Hydroxy phenylacetic acid sulfate | PR |
|  | 2-[4-ethenyl-4-methyl-3-(1- methylethenyl)cyclohexyl]-2- methyl- Oxirane | RR, CR |


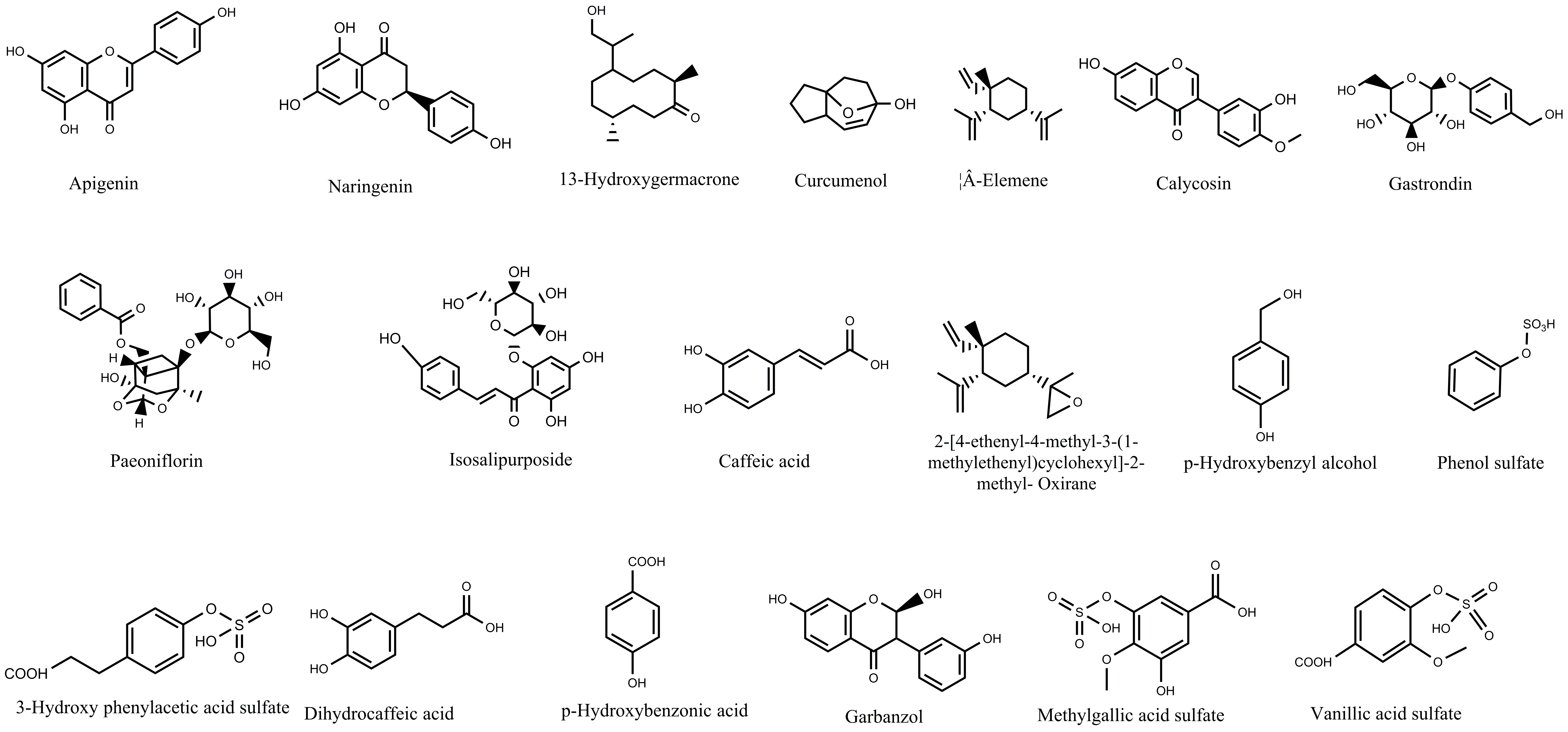


Supplementary Figure 1.The absorbed constituents and metabolites detected in brain


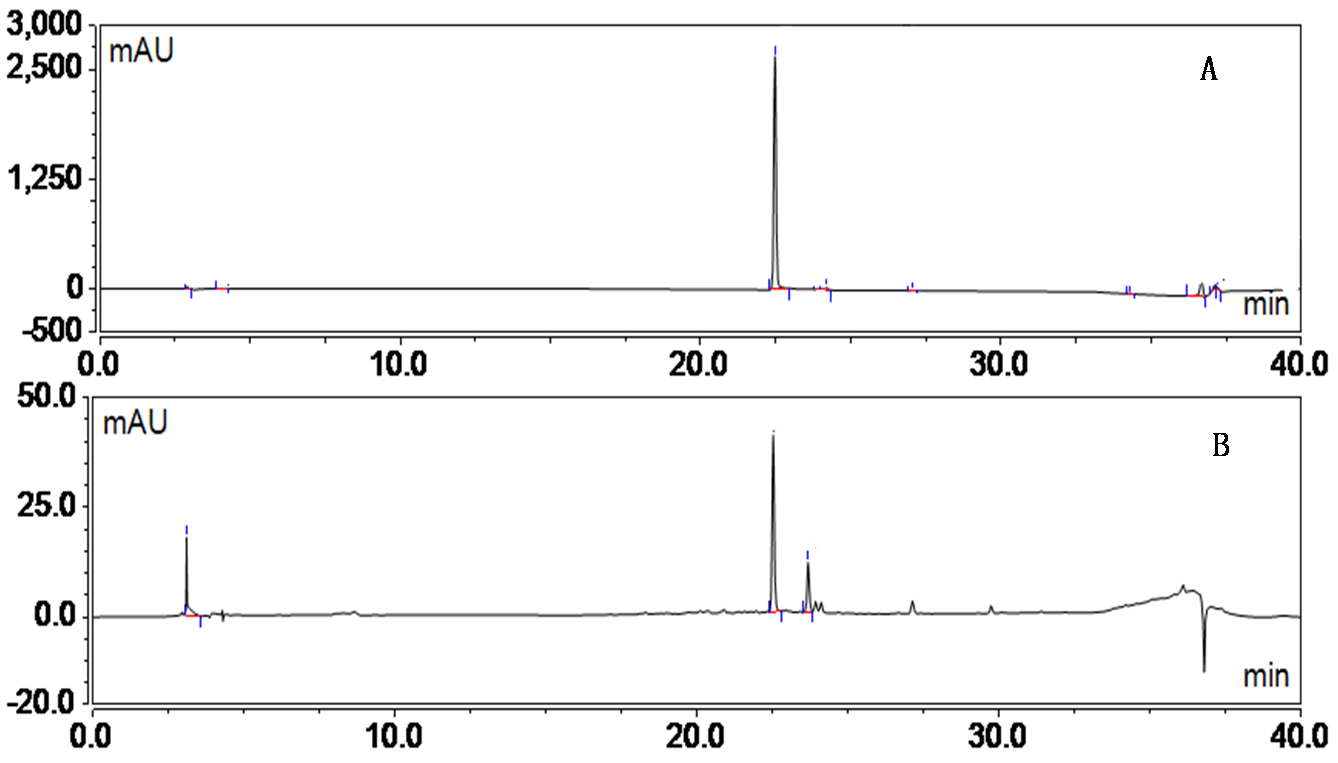


Supplementary Figure 2. (A) Chromatogram of paeoniflorin (B) Chromatogram of NSTC

Supplementary Table 2 Test terms and scoring standards of mNSS

| Exercise test | methodology |
| --- | --- |
| Tail suspension test | The tail of the mouse was fixed and suspended, and the state of the mouse limbs was observed. The forelimb curling was scored 1 point. The hind limb curling was scored 1 point. The head rotation exceeds the central axis by 10 degrees within 30 seconds, which was scored 1 point. Full score is 3 points. |
| Ground placement experiment | The mouse was on the platform and the state was observed. If it walked normally, score 0 points. It walked but unable to walk straight, score 1 point. One side of the body paralyzed, walking in the direction of paralysis was scored 2 points. Tilting toward the direction of paralysis was scored 3 points. Full score is 3 points. |
| Placement test | The mouse was raised by the tail and slowly approached the front table. If the forelimbs could not grab the edge of the table immediately, 0.5 points were scored. The mouse was placed in the bucket so that it could not see and feel the external environment with its tentacles. Its forelimbs were gently touched, with the stimulation only acting on the skin and hair. If there was no response, score 0.5 points. The full score is 1 point. |
| Proprioception test | The mouse was placed in the bucket so that it could not see and feel the external environment with its tentacles. Its forelimbs were pressed forcefully, with stimulating deep muscles and joints. If there was no response, scored 1 point. The full score is 1 point. |
| Balance beam test | The balance beam was a square wooden rod with a length of 80 cm and a width of 2 cm. It was placed flatly at a height of 10 cm from the ground. If the mouse walked steadily above and had a normal posture, it would be scored 0 points. If it held the balance beam but not walking was scored 1 point. If it held the balance beam, but a fall of one limb was counted as 2 points. Two limbs falling or rotating the balance beam (>60s) were counted as 3 points. Trying to maintain balance but falling (>40s) counted as 4 points. Trying to maintain balance but falling (>20s) counted as 5 points. Falling and totally unable to maintain balance or hanging on a balance beam (<20s) would score 6 points. The full score is 6 points. |
| Auricle reflex | The ear canal of the mouse was touched lightly, if it shaked head normally, scored 0 points. If there was no reflection, scored 1 point. |
| Corneal reflex | The cornea touched lightly with a cotton swab, 0 points for blinking, 1 point for no reflex. |
| Scared reflection | Use cardboard to make a brief noise suddenly. If there is a motion response, it was scored 0 point, and no response was scored 1 point. |
| Abnormal behaviors such as epilepsy and paralysis | If the mouse showed seizures, myoclonus, or dystonia, scored 1 point, and score 0 point for normal. |

Supplementary Table 3 Identification of phytochemical components in NSTC and absorbed components in mice tissues

| No. | tR(min) | formula | [M-H]^-^ | | [M+H]^+^ | | major fragment ions in negative mode | major fragment ions in positive mode | identification | source |
| --- | --- | --- | --- | --- | --- | --- | --- | --- | --- | --- |
|  |  |  | meas. | ppm | meas. | ppm |  |  |  |  |
| 1 | 8.52 | C_13_H_18_O_7_ | 285.0982 | 1.7 |  |  | 123.0560 [M-H-Glc]^-^ |  | Gastrodin^*^ | GR |
| 2 | 10.02 | C_8_H_8_O_4_ |  |  | 169.0493 | -1.6 |  | 93.0326 [M+H-CO_2_H-OCH_3_]^+^ | Vanillic acid | PR, TP |
| 3 | 10.25 | C_13_H_16_O_9_ | 315.0721 | 3.1 |  |  | 153.0182 [M–H–Glc]^–^ |  | Protocatechuic acid-3-O-glucoside | PR |
| 4 | 10.32 | C_7_H_6_O_4_ | 153.0199 | 10 | 155.0339 | 0.1 | 91.0203 [M-H-H_2_O-CO_2_]^-^ | 93.0378 [M+H-H_2_O-CO_2_]^+^ | Protocatechuate^*^ | PR |
| 5 | 10.8 | C_21_H_24_O_11_ | 451.1235 | 0 |  |  | 289.0718 [M-H-Glc]^-^ |  | Catechin-7-O-glucoside | PR |
| 6 | 11.43 | C_23_H_28_O_12_ | 495.1501 | 0.8 |  |  | 121.0266 [BA-H]^-^ |  | Hydroxy-paeoniflorin | PR |
| 7 | 11.51 | C_9_H_10_O_5_ | 197.0454 | -0.8 | 199.0601 | 0.2 | 162.83975 [M-H-CO]^-^ | 153.0204, 107.0142 | Syringic acid | PR |
| 8 | 11.72 | C_16_H_18_O_9_ | 353.0878 | 3.2 | 355.1027 | 0.9 | 191.0557, 179.0343 | 164.0402 [M+H-C_10_H_8_O_4_]^+^ | Chlorogenic acid^*^ | PR |
| 9 | 11.95 | C_15_H_14_O_6_ | 289.0713 | 2.4 | 291.0866 | 0.9 | 245.0832 [M-H-CO_2_]^-^ | 139.0441 | Catechin^*^ | PR, TP |
| 10 | 12.10 | C_7_H_6_O_5_ |  |  | 171.0289 | 0.8 |  | 109.0334 [M+H- HCOOH]^+^, | Gallic acid^*^ | PR |
| 11 | 12.48 | C_33_H_40_O_20_ | 755.2059 | 3.9 | 757.21833 | -0.3 | 301.0355 [M-2H-2Rha-Glc]^-^ | 303.0484 [M+2H-2Rha-Glc]^+^ | Quercetin-3-O-(2G-a-L-rhamnosyl)-rutinoside | TP |
| 12 | 12.60 | C_20_H_28_O_12_ | 459.1501 | 0.9 |  |  | 165.0548 [M-H-Ara-Glc]^-^ |  | Paeonolide | PR |
| 13 | 12.91 | C_33_H_40_O_19_ | 739.2083 | 0.5 |  |  | 284.0325 [M-2H-2Rha-Glc]^-^, |  | Kaempferol-3-O-(2G-a- L-rhamnosyl)-rutinoside | TP |
| 14 | 12.96 | C_34_H_42_O_20_ | 769.2217 | 4.2 | 771.2339 | -0.4 | 315.0506[M-H-2Rha-Glc]- | 317.0636[M+H-2Rha-Glc]^+^ | Typhaneoside^*^ | TP |
| 15 | 13.01 | C_27_H_30_O_16_ | 609.1459 | 1.4 | 611.1613 | 1 | 300.0290 [M-H-Rha-Glc]^-^, | 303.0439 [M+2H-Rha-Glc]^-^, | Quercetin-3-O-neohesperidoside | TP |
| 16 | 13.08 | C_23_H_28_O_11_ | 479.1555 | 1.6 |  |  | 165.0560 [M-H-BA-Glc]^-^ 327.1110 [M-H-BA]^-^ |  | Paeoniflorin^*^ | PR |
| 17 | 13.11 | C_34_H_28_O_22_ | 787.1022 | 4.1 |  |  | 618.0911 [M-GA]^-^, |  | 2,3,4,6-Tetra-O-galloyl-*β*-glucose | PR |
| 18 | 13.15 | C_21_H_20_O_12_ | 463.0873 | 0.6 | 465.1030 | 0.5 | 301.0874 [M-H-Glc]^-^ | 303.0157 [M-H-Glc]^+^ | Quercetin-3-glucoside | TP, GR |
| 19 | 13.51 | C_41_H_32_O_26_ | 939.1111 | 1.4 |  |  | 769.0969 [M-H-GA]^-^,  617.0823 [M-H-GA-C_7_H_4_O_4_]^-^, |  | 1,2,3,4,6-Penta-O-galloyl-*β*-glucose | PR, RR |
| 20 | 13.58 | C_28_H_32_O_16_ | 623.1617 | 1.6 | 625.1765 | 0.4 | 314.0517 [M-H-Glc-Rha]^-^ | 317.0689[M+2H-Glc-Rha]^+^ | Isorhamnetin-3-O-rutinoside | CR, TP |
| 21 | 13.62 | C_27_H_44_O_7_ |  |  | 481.3164 | 0.9 |  | 371.2212 [M+H-3H_2_O-C_4_H_8_]^+^ | β-Ecdysterone^*^ | RR |
| 22 | 13.65 | C_16_H_12_O_5_ | 283.0614 | 4.4 | 285.0755 | -1 | 268.0352 [M-H-CH_2_]^-^ | 270.0511 [M+H–CH_3_]^+^ | Calycosin^*^ | CR |
| 23 | 13.85 | C_27_H_30_O_15_ | 593.151 | -0.4 |  |  | 285.0425 [M-H–Rha–Glc]^-^ |  | Kaempferol -3-O-neohesperidin | TP |
| 24 | 13.89 | C_28_H_32_O_16_ | 623.1606 | -0.1 |  |  | 314.0415 [M-H-Glc-Rha]^-^ |  | Isorhamnetin-3-O-neohespeidoside^*^ | CR, TP |
| 25 | 13.9 | C_30_H_32_O_15_ | 631.1662 | 0.7 |  |  | 613.1613 [M-H-H_2_O]^-^, |  | Galloylpaeoniflorin | GR, PR |
| 26 | 14.4 | C_22_H_22_O_12_ | 477.1021 | 0 | 479.1189 | 1 | 315.0513 [M-H-Glc]^-^, | 317.0633 [M+H-Glc]^+^ | Isorhamnetin-3-O-glucopyranoside | PR, RR , TP |
| 27 | 14.53 | C_21_H_22_O_10_ | 433.113 | -2.5 | 435.1283 | -0.7 | 271.0613 [M-H-Glc]^-^ | 273.0771 [M+H-Glc]^+^ | Isosalipurposide | PR |
| 28 | 14.67 | C_21_H_18_O_11_ | 446.08 | 1 | | 447.0917 | | --- | | -1.2 | 269.0449 [M-H-Glucuronide]^-^ | 271.0555 [M+H-Glucuronide]^+^, | Apigenin-7-O-*β*-glucuronide | PR |
| 29 | 15.32 | C_30_H_32_O_12_ | 583.1814 | 0.6 |  |  | 431.1323 [M-H-BA-HCOOH]^-^ |  | Benzoyl paeoniflorin | PR |
| 30 | 15.54 | C_30_H_32_O_13_ | 599.177 | 1.8 |  |  | 431.1378 [M-H-H_2_O-HCOH-C_7_H_4_O_2_]^-^ |  | Benzoyl oxypaeoniflorin | PR |
| 31 | 18.54 | C_16_H_12_O_7_ | 315.0508 | 2.5 |  |  | 300.0294 [M-H-CH_3_]^-^ |  | Isorhamnetin | PR |
| 32 | 18.78 | C_15_H_12_O_5_ | 271.0612 | 2.8 | 273.0758 | 0.2 | 151.0022 [M-H-C_8_H_8_O]^-^ | 153.0571 [M+H-C_8_H_8_O]^+^ | Naringenin^*^ | PR, TP |
| 33 | 18.93 | C_15_H_10_O_5_ | 269.0456 | 1 | 271.0603 | 0.8 | 107.0146 [M-H-Glc]^-^, | 153.0184 [M-H-C_8_H_8_O]^+^ | Apigenin^*^ | PR |
| 34 | 20.61 | C_13_H_10_OS_2_ |  |  | 247.0247 | 0.3 |  | 205.0145 [M+H-CO_2_]^+^, | Arctinone b | RR |
| 35 | 20.66 | C_15_H_18_O_2_ |  |  | 231.1381 | 0.7 |  | 185.1309 [M+H-C_2_H_4_-H_2_O]^+^ | Epi-curzerenone | CR |
| 36 | 21.54 | C_15_H_24_O_2_ |  |  | 237.1845 | -1.6 |  | 191.0925 [M+H-CH_2_O_2_]^+^, | Curdione | RR |
| 37 | 21.84 | C_15_H_22_O_2_ |  |  | 235.1691 | -0.7 |  | 179.1174 [M+H–O]^+^, | 13-Hydroxygermacrone | CR |
| 38 | 22.47 | C_15_H_16_O_2_ |  |  | 229.1220 | -1.2 |  | 159.0812 [M+H-CO-CH_2_-H_2_O]^+^ | (+)-Curzeone | CR |
| 39 | 23.33 | C_12_H_8_O_2_S_2_ |  |  | 249.0036 | -0.9 |  | 205.0144 [M+H-CO_2_]^+^, | Arctic acid | RR |
| 40 | 24.07 | C_15_H_24_O_2_ | 246.9886 | 1.7 | 237.1847 | -0.6 | 202.9955 [M-H-CO_2_]^-^ | 149.1347 [M+H-CH_2_O_2_-COOH]^+^ | Curcumenol | CR |
| 41 | 26.44 | C_15_H_20_O |  |  | 217.1587 | -0.1 |  | 199.1494 [M+H-H_2_O]^+^, | Curzerene | CR |
| 42 | 27.43 | C_15_H_22_O |  |  | 219.1745 | 0.8 |  | 91.0552 [M+H-O-2H-C_9_H_12_]^+^, 159.1202 | Germacrone | CR, PR |
| 43 | 31.14 | C_15_H_24_ |  |  | 205.1948 | -1.4 |  | 107.0901 [M+H-C_7_H_13_]^+^ | *β*-Elemene^*^ | CR, RR |

Footnote: ^*^Compared with reference standards; TP, Typhae Pollen; PR, Radix Paeoniae Rubra; CR, Rhizoma Gastrodiae, GR, Radix Rhapontici; RR, Radix Curcumae; BA, benzoic acid; GA, gallic acid.

Supplementary Table 4 Topology characteristics of hub nodes from PPI network

|  | Degree | Betweemness centrality | Center closeness |
| --- | --- | --- | --- |
| INS | 81 | 0.124388 | 0.75159236 |
| AKT1 | 75 | 0.054628 | 0.72839506 |
| IL6 | 72 | 0.05921 | 0.71084337 |
| TP53 | 70 | 0.053966 | 0.70658683 |
| TNF | 69 | 0.053901 | 0.69822485 |
| VEGFA | 63 | 0.036124 | 0.67045455 |
| JUN | 60 | 0.019438 | 0.66292135 |
| CASP3 | 60 | 0.031316 | 0.66292135 |
| MAPK1 | 59 | 0.017711 | 0.65921788 |
| PTGS2 | 56 | 0.025136 | 0.64480874 |
| FOS | 56 | 0.050491 | 0.64480874 |
| EGFR | 55 | 0.01422 | 0.64130435 |
| SRC | 53 | 0.031344 | 0.6344086 |
| HSP90AA1 | 51 | 0.013994 | 0.6344086 |
| CCND1 | 51 | 0.031137 | 0.62765957 |
| MMP9 | 49 | 0.010076 | 0.62105263 |
| BCL2L1 | 49 | 0.011519 | 0.61780105 |
| ESR1 | 47 | 0.009151 | 0.61139896 |
| NOS3 | 44 | 0.004763 | 0.60824742 |
| MAPK14 | 44 | 0.040061 | 0.60512821 |
| IL4 | 42 | 0.01736 | 0.5959596 |
| IL2 | 41 | 0.008178 | 0.59 |
| HMOX1 | 39 | 0.021875 | 0.59 |
| NR3C1 | 38 | 0.003016 | 0.58415842 |
| MCL1 | 38 | 0.007387 | 0.58415842 |
| CDKN1A | 38 | 0.010213 | 0.58128079 |
| ICAM1 | 37 | 0.004494 | 0.58128079 |
| HIF1A | 37 | 0.004956 | 0.58128079 |
| AR | 37 | 0.011533 | 0.57843137 |
| MDM2 | 36 | 0.006049 | 0.57560976 |
| HGF | 34 | 0.002233 | 0.57560976 |
| CASP9 | 34 | 0.002683 | 0.57281553 |
| NFKB1 | 33 | 0.001043 | 0.56190476 |
| IGF1R | 32 | 0.001576 | 0.56190476 |
| NFKBIA | 30 | 9.62E-04 | 0.56190476 |
| MAPK10 | 30 | 0.005947 | 0.55399061 |
| PGR | 29 | 0.001792 | 0.55399061 |
| IL13 | 29 | 0.006011 | 0.55140187 |
| CD40LG | 28 | 0.001963 | 0.55140187 |
| RAF1 | 27 | 0.00131 | 0.54883721 |
| NOS2 | 27 | 0.002196 | 0.5462963 |
| LCK | 27 | 0.002672 | 0.5437788 |
| F2 | 27 | 0.012256 | 0.5437788 |
| MMP3 | 26 | 3.48E-04 | 0.5437788 |
| MMP1 | 26 | 3.56E-04 | 0.5412844 |
| HSPA8 | 26 | 0.007174 | 0.5412844 |
| PTPN1 | 25 | 0.005624 | 0.53636364 |
| SLC2A4 | 24 | 0.004052 | 0.53636364 |
| ALOX5 | 23 | 0.007947 | 0.53636364 |
| NQO1 | 22 | 0.002573 | 0.53393665 |
| IKBKG | 22 | 0.003986 | 0.53393665 |
| ADRB2 | 22 | 0.006955 | 0.53153153 |
| ABCG2 | 22 | 0.009657 | 0.52444444 |
| NOS1 | 21 | 0.001051 | 0.52212389 |
| NFE2L2 | 21 | 0.001298 | 0.51754386 |
| CHEK1 | 21 | 0.019967 | 0.51528384 |
| PPARA | 18 | 8.44E-04 | 0.51528384 |
| CCND2 | 18 | 0.002148 | 0.51304348 |
| BCL2 | 18 | 0.017305 | 0.51082251 |
| GSTP1 | 17 | 7.53E-04 | 0.50643777 |
| CTSB | 17 | 0.002503 | 0.50212766 |
| PLK1 | 16 | 0.001842 | 0.5 |
| MAPKAPK2 | 15 | 0.003429 | 0.5 |
| CFTR | 15 | 0.011077 | 0.49579832 |
| MME | 14 | 2.30E-04 | 0.49579832 |
| CYP2C9 | 14 | 0.001253 | 0.49579832 |
| BAX | 14 | 0.003144 | 0.49579832 |
| PTGS1 | 13 | 1.85E-04 | 0.49372385 |
| NR3C2 | 13 | 6.01E-04 | 0.49372385 |
| MAPK12 | 13 | 6.82E-04 | 0.48962656 |
| INSR | 13 | 0.001759 | 0.48962656 |
| DPP4 | 13 | 0.002222 | 0.48760331 |
| DHFR | 13 | 0.013814 | 0.48559671 |
| SERPINC1 | 12 | 1.30E-05 | 0.48360656 |
| MMP8 | 12 | 0.001348 | 0.48360656 |
| HPRT1 | 12 | 0.002311 | 0.48163265 |
| PCK1 | 11 | 3.44E-04 | 0.48163265 |
| FABP4 | 11 | 0.001171 | 0.4796748 |
| BCHE | 11 | 0.001802 | 0.4796748 |
| TXNRD1 | 10 | 4.56E-04 | 0.4796748 |
| FKBP1A | 10 | 6.80E-04 | 0.47773279 |
| ADRB1 | 10 | 7.64E-04 | 0.47580645 |
| ITGAL | 9 | 1.33E-04 | 0.47389558 |
| IFNGR2 | 9 | 1.36E-04 | 0.472 |
| IFNGR1 | 9 | 1.98E-04 | 0.472 |
| DRD1 | 9 | 0.003118 | 0.47011952 |
| CHRM1 | 9 | 0.004569 | 0.46825397 |
| PLA2G2A | 8 | 1.31E-04 | 0.46640316 |
| HMGCR | 8 | 3.12E-04 | 0.46640316 |
| CYP2C19 | 8 | 4.29E-04 | 0.46640316 |
| ANG | 8 | 5.88E-04 | 0.45736434 |
| ADRA1D | 8 | 5.95E-04 | 0.45736434 |
| ADRA1A | 8 | 5.95E-04 | 0.45559846 |
| ACE2 | 8 | 6.27E-04 | 0.45559846 |
| TK1 | 7 | 5.51E-05 | 0.45384615 |
| RBL2 | 7 | 0.003134 | 0.45384615 |
| GRIA2 | 7 | 0.004749 | 0.4486692 |
| CD14 | 6 | 9.74E-05 | 0.4486692 |
| ADRA2A | 6 | 1.13E-04 | 0.4486692 |
| F7 | 5 | 0 | 0.4486692 |
| CES1 | 5 | 6.86E-05 | 0.44360902 |
| CA2 | 5 | 8.43E-05 | 0.44194757 |
| ALPI | 5 | 1.39E-04 | 0.44194757 |
| ALOX5AP | 5 | 2.09E-04 | 0.43065693 |
| ADRA2C | 5 | 3.92E-04 | 0.42909091 |
| KMT2A | 4 | 0 | 0.42753623 |
| HSD11B1 | 4 | 3.62E-05 | 0.42446043 |
| GABRA1 | 4 | 2.56E-04 | 0.41696113 |
| FCER2 | 3 | 0 | 0.41403509 |
| ATP5B | 3 | 6.37E-04 | 0.4 |
| SHMT1 | 2 | 0 | 0.39864865 |
| MAPK4 | 2 | 0 | 0.39864865 |
| CAMK1 | 2 | 2.79E-05 | 0.38943894 |
| CA4 | 2 | 1.24E-04 | 0.38943894 |
| ALAD | 2 | 1.52E-04 | 0.37579618 |
| SCN5A | 1 | 0 | 0.36085627 |
| GFER | 1 | 0 | 0.35014837 |
| GAA | 1 | 0 | 0.32777778 |
| CA3 | 1 | 0 | 0.32777778 |

Supplementary Table 5 The common signaling pathways shared by 138 putative targets and differentially expressed genes at 4h, 24h, 7d after ischemic stroke

| time point | pathway |
| --- | --- |
| 4h after ischemia stroke | Neuroactive ligand-receptor interaction |
|  | Calcium signaling pathway |
|  | cAMP signaling pathway |
|  | MAPK signaling pathway |
|  | PI3K-Akt signaling pathway |
|  | Adrenergic signaling in cardiomyocytes |
|  | Malaria |
|  | Pathways in cancer |
|  | TNF signaling pathway |
|  | FoxO signaling pathway |
|  | Amphetamine addiction |
|  | Focal adhesion |
|  | Cocaine addiction |
|  | Small cell lung cancer |
|  | Toxoplasmosis |
|  | Osteoclast differentiation |
|  | Hematopoietic cell lineage |
|  | GnRH signaling pathway |
|  | Ras signaling pathway |
|  | p53 signaling pathway |
|  | HIF-1 signaling pathway |
|  | Apoptosis |
|  | Hepatitis B |
|  | Glioma |
|  | MicroRNAs in cancer |
|  | Dopaminergic synapse |
|  | Toll-like receptor signaling pathway |
|  | NF-kappa B signaling pathway |
|  | Chemokine signaling pathway |
|  | Proteoglycans in cancer |
|  | T cell receptor signaling pathway |
| 24h after ischemia stroke | Malaria |
|  | Neuroactive ligand-receptor interaction |
|  | TNF signaling pathway |
|  | Pertussis |
|  | Amphetamine addiction |
|  | Salivary secretion |
|  | NOD-like receptor signaling pathway |
|  | Toll-like receptor signaling pathway |
|  | Rheumatoid arthritis |
|  | Chemokine signaling pathway |
|  | Adrenergic signaling in cardiomyocytes |
|  | Osteoclast differentiation |
|  | Tuberculosis |
|  | Gap junction |
|  | cAMP signaling pathway |
| 7d after ischemia stroke | Osteoclast differentiation |
|  | MAPK signaling pathway |
|  | Transcriptional misregulation in cancer |
|  | Intestinal immune network for IgA production |
|  | FoxO signaling pathway |
|  | Amphetamine addiction |
|  | T cell receptor signaling pathway |
|  | Amoebiasis |
|  | Neuroactive ligand-receptor interaction |
